# Wellbeing status and priority concerns of patients with advanced renal cell carcinoma: results of the EONS PROMs project international online survey

**DOI:** 10.1007/s00520-025-09585-5

**Published:** 2025-06-03

**Authors:** Grigorios Kotronoulas, Celia Diez de los Rios de la Serna, Amanda Drury, Wendy H. Oldenmenger, Daniel Kelly

**Affiliations:** 1 School of Medicine, Dentistry & Nursing, University of Glasgow, Glasgow, UK; 2 University of Glasgow, Glasgow, UK; 3 European Oncology Nursing Society, Brussels, Belgium; 4 Dublin City University, Dublin, Ireland; 5 Erasmus MC, University Medical Center, Rotterdam, The Netherlands; 6 Cardiff University, Cardiff, UK

**Keywords:** Renal cell carcinoma, Wellbeing, Priority concerns, Impact, Patient-reported outcomes, Supportive care

## Abstract

**Purpose:**

Living with advanced renal cell carcinoma (RCC) can be challenging. Previous research suggests that patients are faced with variable complexities, although the main focus has been on physical problems. We aimed to generate empirical evidence to better understand patients’ perceptions of adverse impact on wellbeing, to reveal priority concerns, and to explore moderators that could point to a greater risk for declining health status in this patient population.

**Methods:**

A prospective, international, and cross-sectional online survey was conducted, comprising a demographic/clinical data form, the Functional Assessment of Cancer Therapy-Biologic Response Modifiers (FACT-BRM) questionnaire, and bespoke closed- and open-ended questions.

**Results:**

Data from 105 participants were analysed. The typical participant was male, on targeted therapy, and middle-aged (median 42 years), with a median of 54 months since diagnosis, and predominantly originated from the USA or UK. Being unable to work (46%), worrying that their condition would worsen (45%), concerns about psychological support for their partner or family (44%), and being burdened by urinary frequency (43%) were major problems for over 40% of this sample. Concerns about future response to treatment, running out of treatment options, cancer relapse, declining health, dying, and impact on family were also expressed. Older age was linked to higher wellbeing scores.

**Conclusion:**

Relying on patient-reported outcomes, we were able to reveal the impact of advanced RCC and its management on several interrelated areas of patient wellbeing. These findings need to be validated in other contexts to ensure they are generalisable.

**Supplementary Information:**

The online version contains supplementary material available at 10.1007/s00520-025-09585-5.

## Introduction

Renal cell carcinoma (RCC) is the most common type of kidney cancer, accounting for 80–85% of all kidney cancers [1]. Approximately 30% of RCC cases are diagnosed as metastatic (TNM stage 4), and up to 50% progress to an advanced stage (TNM stage ≥ 3) despite or before surgical removal of the primary tumour [2, 3]. This makes metastatic RCC the deadliest urological cancer, with a mere 12% of patients surviving beyond 5 years [3].

Living with advanced RCC can be challenging. Emerging evidence suggests that patients are faced with the complexities of an advanced cancer diagnosis, comorbid paraneoplastic syndromes, and challenging physical and psychosocial issues [4–6]. Consequently, patients may have to make difficult decisions about treatment, supportive care, and palliative care that impact their wellbeing and that of their family [4, 7] as well as increasing demand on healthcare services [5].

The intensity of the experience of advanced (renal cell) cancer necessitates regular and structured assessments of patients’ wellbeing. This is to ensure timely management of deteriorating health and focus of tailored support offered for burdensome issues that the patient indicates as priority. Such evaluation is best facilitated by measuring patient-reported outcomes (PROs) via patient-reported outcome measures (PROMs) [8, 9]. The use of PROMs in cancer practice and research has gained considerable traction over the past 10 years [9, 10], with PRO data now considered key indicators of patient benefit in cancer care [10].

Until recently, the PROs that should be considered as core in advanced RCC were not clear [11]. As part of a larger project (https://cancernurse.eu/research/proms_project/), our team established a core set of 49 PROs for consideration of evaluation in advanced RCC [12]. Once having this key knowledge, we aimed to generate additional empirical evidence to help better understand patients’ perceptions of adverse impact on core PROs, reveal priority concerns, and explore moderators of wellbeing, which could point to groups at greater risk for declining PROs associated with advanced RCC. Results of this investigation are reported here. Specifically, we sought to address this primary question:

- What is the level and extent of impact on core PROs in advanced RCC in terms of patient-reported incidence and/or severity?

Secondarily, we analysed data to answer two questions:

- When spontaneously asked, which core PROs do patients with advanced RCC consider a priority?
- Which demographic or clinical variables are linked to better or worse wellbeing in advanced RCC?

## Methods

### Study design

A prospective, international, and cross-sectional online survey design was employed, comprising both closed- and open-ended questions [13]. Reporting is in line with the Consensus-Based Checklist for Reporting of Survey Studies (CROSS) [14].

### Sampling considerations

Patients were eligible for participation if they were adults (≥ 18 years), self-identified as living with advanced RCC (TNM stages 3 and 4 [15]), and able to write and understand English. Patients without Internet access and unable to provide consent to the online survey could not participate.

Calculations were based on current RCC incidence worldwide [16] and prevalence rates of advanced RCC (20–33%) [17, 18], which yielded a target population of approximately 107,000 people living with RCC. We anticipated that the accessible population would likely be 1/100 of the target population, i.e. approximately 1100 patients with advanced RCC. With a sampling confidence level set at 95% and a margin of error set at 7–10%, a sample size of 89–167 patients with advanced RCC would be required (https://www.surveymonkey.com/mp/sample-size-calculator/).

### Recruitment procedures

Patients were identified internationally via several sources, including advertisements on professional and charitable organisations via Twitter, Facebook, and LinkedIn, direct invitations to collaborating patient support groups, and a snowballing technique, whereby participants were asked to identify additional potential participants from their own networks. Regular reminders were sent out on a 3-weekly basis.

### Data collection

The survey was set up on the Online Surveys tool (https://www.onlinesurveys.ac.uk/) and piloted for functionality. A survey link was created, ready for circulation. The survey link introduced potential participants to the study and directed them to the participant information sheet. A demographic/clinical data form asked about gender, age, country of residence, date of diagnosis, and current treatment.

The Functional Assessment of Cancer Therapy-Biologic Response Modifiers (FACT-BRM), version 4 [19] PROM was selected as most suitable in this research. The internal consistency, sensitivity to change, and concurrent and discriminant validity of the FACT-BRM have been confirmed in previous research [19, 20]. The FACT-BRM comprises 40 items with a recall period of the past 7 days. Each item is measured on a 5-option numerical scale (0—not at all, 1—a little bit, 2—somewhat, 3—quite a bit, 4—very much). FACT-BRM items are split into the following subscale domains: physical wellbeing (PWB), social/family wellbeing (SWB), emotional wellbeing (EWB), functional wellbeing (FWB), BRM-physical, and BRM-mental. Three total scores can be calculated: FACT-BRM Trial Outcome Index (TOI), FACT-General (FACT-G), and FACT-BRM [19]. Higher scores indicate better wellbeing.

To account for core PROs not addressed by the FACT-BRM, 30 additional items were developed and discussed with our steering group for relevance and wording (Supplementary file 1). For consistency, these additional items were measured using the same 5-option numerical scale as for the FACT-BRM.

Finally, the following three open-ended questions (OEQs) were developed and included in the survey to gain a greater understanding of patients’ perceptions of priority PROs and support with reported needs:

- OEQ 1: Are there any other issues that we have not mentioned but are important to you?
- OEQ 2: Is there any help from your clinical team that you need but have not received yet?
- OEQ 3: Please tell us the 3 things that you are most concerned about.

### Data management and analysis

Data were first entered in an Excel spreadsheet and visually inspected for errors and missing values. Missing data were only minimal (63 out of 7350 expected, 0.85%), and most (*n* = 50) referred to one participant. Missing data were highlighted but not substituted. Frequency count calculation disregarded items with missing data, and the total of participants was adjusted accordingly. Scale score calculation also disregarded items with missing data as per scoring instructions.

We conducted univariate analyses on all data. Data from the OEQs were content analysed and tabulated. Each participant’s responses on the OEQs were read, and short labels (codes) were applied to reflect the content. Codes of similar content were grouped together to create a category, including the number of codes within each category [21]. For continuous and interval-scale demographic and clinical variables (i.e. age, time since diagnosis), we computed the mean, standard deviation (SD), and range. We generated frequency counts for categorical demographic and clinical variables (gender, country of origin, type of treatment) and individual FACT items to describe response patterns (*n*, %). In all analyses, positively worded individual items were reversed so that scoring was consistent across all individual items and to allow for interpretation. Although we used the original FACT 5-option response format, for graphical representation and visualisation purposes, item response was collapsed into 3 categories, i.e. 0–1 = not a problem, 2 = somewhat of a problem/moderate problem, and 3–4 = a major problem.

Subscale scores (PWB, SWB, EWB, FWB, BRM-physical, BRM-mental) and total scores for FACT-BRM TOI, FACT-G, and FACT-BRM were calculated and summarised via the use of median, mean, standard deviation, and range (Supplementary file 2). Cronbach’s alphas were calculated for the six subscales to indicate the level of internal consistency reliability. Cronbach’s alphas ranged from 0.76 (EWB) to 0.88 (PWB), all above the 0.70 benchmark for acceptable reliability [22]. We used Q-Q plots, histograms, and the Kolmogorov–Smirnov test to check the assumption of normality in subscale and total scores. We noted no major deviations from normality.

To address our secondary objective, we used linear regression analysis to examine the association between demographic/clinical variables (i.e. gender, age, type of treatment, time since diagnosis) and subscale/total FACT scores. Data on age and time since diagnosis were checked with no major deviations from normality. Country of origin was not considered due to too few cases across several categories. To include type of treatment in the models, we created four dummy variables (reference category: chemotherapy). We investigated assumptions relating to the normal distribution of errors and multicollinearity; no modifications to the analysis were required. Individual multivariate linear regression models were created for each of the following dependent variables: PWB, SWB, EWB, FWB, FACT-BRM TOI, FACT-G, and FACT-BRM. We set the level of significance at 0.05. The 95% confidence intervals (CI) for standardised regression coefficients were reported. IBM SPSS (IBM Inc. Chicago, IL) aided the statistical analysis.

### Ethics

The study was conducted in accordance with the Declaration of Helsinki. Ethical approval was sought from the University of Glasgow’s School of Medicine, Veterinary and Life Sciences ethics committee (Ref. no: 200200106). Electronic consent was captured, whereby potential participants with the survey link were provided with access to the participant information sheet and, on the next screen, were asked to complete and submit the electronic consent form if they agreed to statements related to procedures of this research. All data (personal and research) were treated as strictly confidential. Research data (i.e. survey results and demographic characteristics) were pseudonymised via the use of participant identification numbers, while identifiable personal data and research data were entered into separate password-protected files that were uploaded on secure University of Glasgow servers.

## Results

### Accrual rates

The survey opened in May 2022 and remained open for 23 weeks. In total, 109 individuals responded, for an average recruitment rate of 4–5 new participants per week. Four individuals did not provide consent and exited the survey. As such, we analysed data from 105 participants with complete responses.

### Sample characteristics

Typically, survey participants were men (61%), on targeted therapy or immunotherapy (31% and 30%, respectively), and middle-aged (median 42 years). The median time since diagnosis was 54 months. Predominantly, participants originated from the USA or UK (Table 1).

**Table 1:** Sample demographic/clinical characteristics (*n* = 105)

	Median	Mean	SD	Min–max
Age (years)	42	43.9	14.1	23–77
Time since diagnosis (years)	54	71.3	56.2	11–313
			*n*	%
Gender				
Male			62	61.4
Age (years)				
18–29			14	13.3
30–39			33	31.4
40–49			22	21.0
50–59			19	18.1
60–69			11	10.5
70 +			6	5.7
Months (years) since diagnosis				
1–12 (≤ 1 year)			1	1.0
13–24 (1–2 years)			11	10.5
25–48 (2–4 years)			36	34.3
49–96 (4–8 years)			35	33.3
97–192 (8–16 years)			13	12.4
193 + (16 + years)			9	8.6
Treatment (*n* = 100)				
Targeted therapy			31	31.0
Immunotherapy			30	30.0
Chemotherapy			21	21.0
Hormone therapy			7	7.0
Radiotherapy			7	7.0
Combined immunotherapy and targeted therapy			2	2.0
No treatment			2	2.0
Country where participant resides				
USA			56	53.3
UK			32	30.5
Japan			5	4.8
Canada			3	2.8
Other (Azerbaijan, Belgium, China, Colombia, Ireland, Isle of Man, Pakistan, Portugal, South Africa)			9	8.6

### Top reported problems

**Fig. 1:**
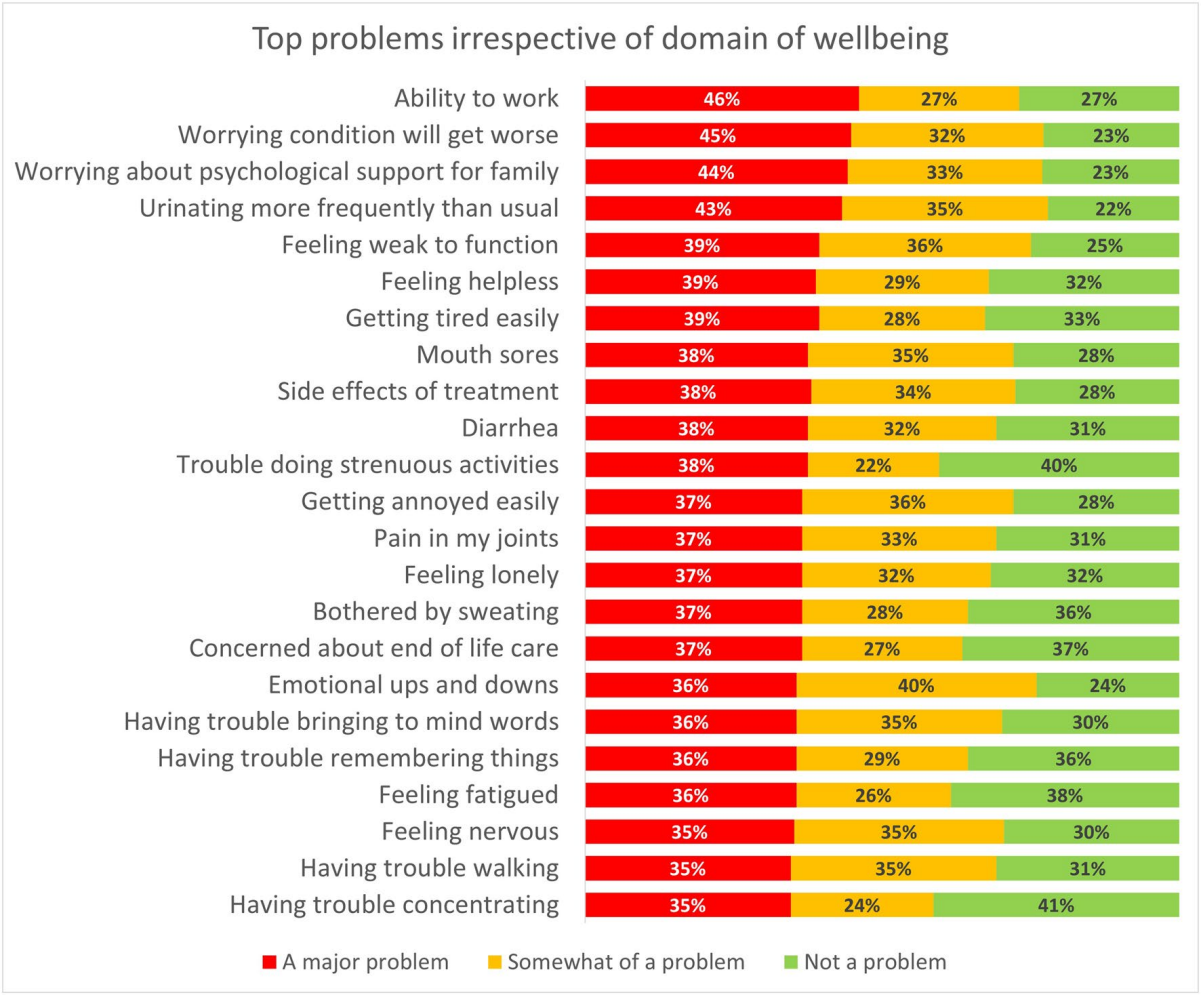
Top problems irrespective of domain of wellbeing. For each item, percentages indicate a major problem (red), somewhat of a problem (yellow), or no particular problem (green). Items show in descending order from most to least frequent ‘major problem’. Items showing in descending order from most to least frequent problem

Across all domains of wellbeing, at least 4 in 10 patients reported ‘a major problem’ because of being unable to work (46%), worrying their condition would worsen (45%), being concerned about the psychological support their partner or family were receiving (44%), and being burdened by too frequent an urge to urinate (43%) (Fig. 1). Additional ‘major problems’ included getting tired easily (39%), feeling weak (39%), and feeling helpless (39%). Urinating more frequently than usual, worrying the condition will get worse, worrying about the psychological support of family, dealing with emotional ups and downs, and feeling weak to function were reported as at least ‘moderate problems’ by at least three quarters of the sample (Supplementary file 3).

### Reported problems per domain of wellbeing

**Fig. 2:**
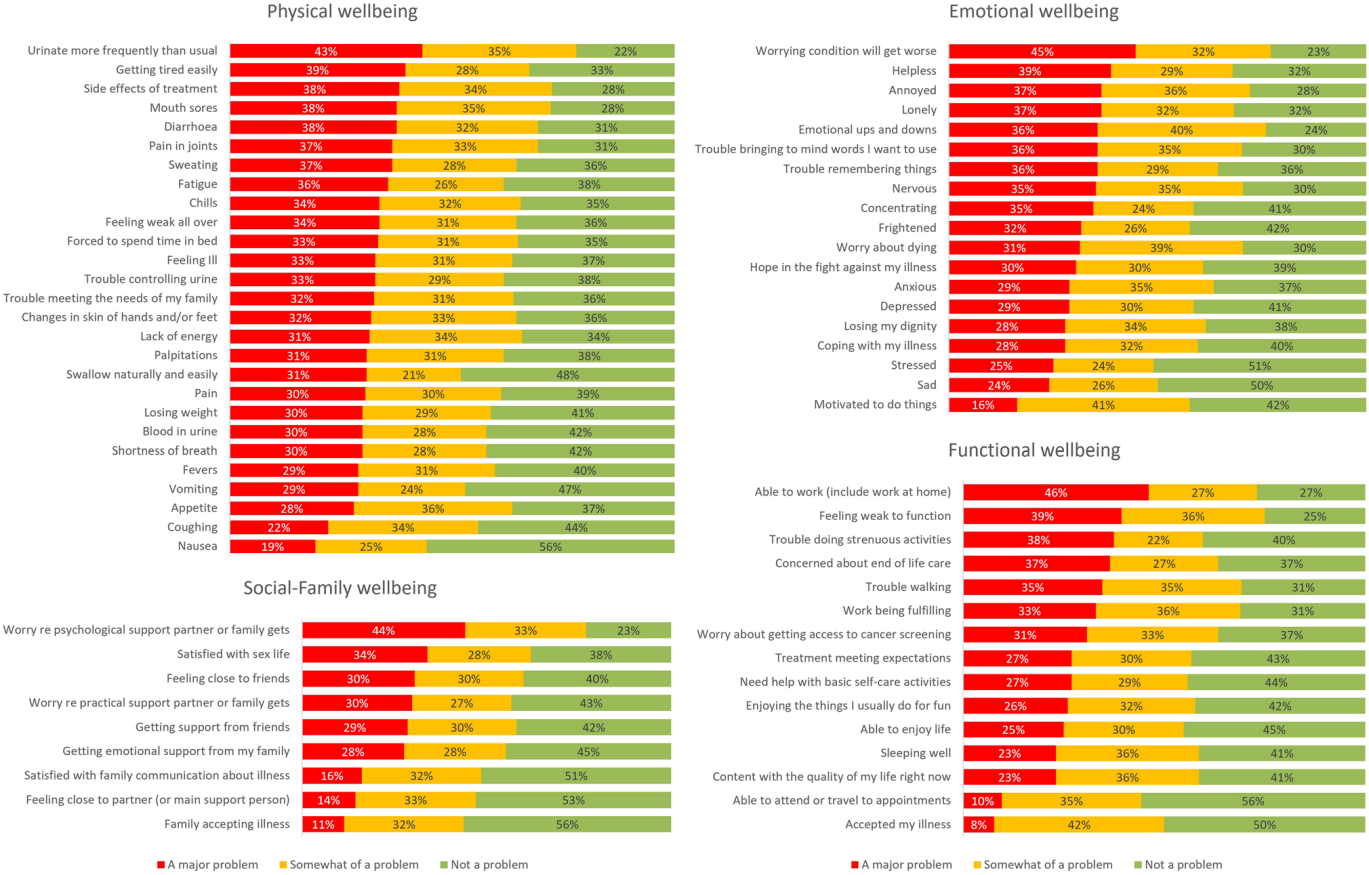
Breakdown of participant responses on individual items for physical wellbeing, emotional, social, and functional wellbeing. For each item, percentages indicate a major problem (red), somewhat of a problem (yellow), or no particular problem (green). Items are shown in descending order from most to least frequent problem

Top major problems per domain are shown in red in Fig. 2. Urinating more frequently than usual, being bothered by the side effects of treatment, dealing with mouth sores, diarrhoea, pain in the joints, and getting tired easily were at least ‘moderate problems’ that affected two-thirds of the sample. Worrying about the psychological support that the family received was reported as at least moderate by over 75% of the sample. Over 60% reported at least moderate problems with their sex life. Within the domain of emotional wellbeing, 7 in 10 participants reported at least moderate issues with worries about dying, cognitive performance, and loneliness. In terms of functional wellbeing, over 70% reported at least moderate issues because of feeling weak to function and being unable to work. Six in 10 participants reported at least moderate concerns with end-of-life care (see details in Supplementary file 3).

### ‘In your own words’ expressed concerns

**Fig. 3:**
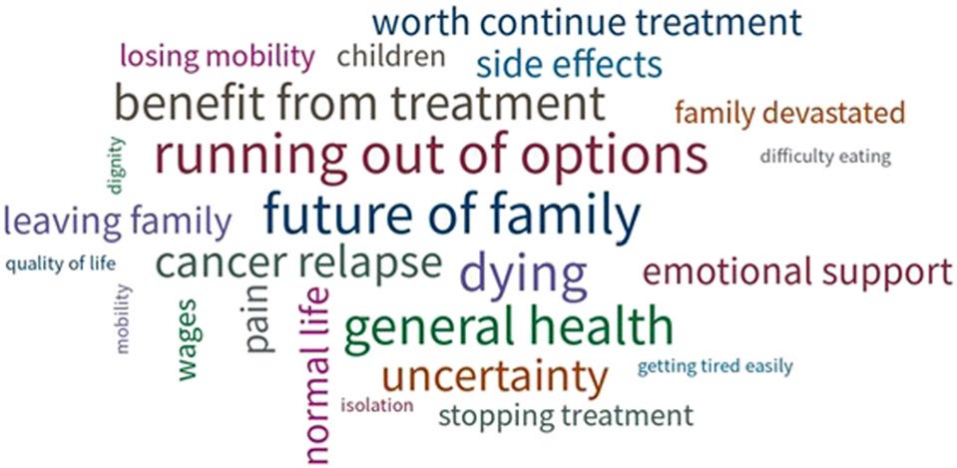
Word cloud of top concerns spontaneously expressed in response to the open-ended question: *Please tell us the 3 things that you are most concerned about.* (*N* = 40) The larger the font size, the more frequent the concern

In response to OEQ 1, 18 participants (17%) identified additional concerns in five different areas, namely in relation to treatment efficacy, availability, and intensity, accessibility to the healthcare system, practicalities of everyday living, peer support and access to psychological support, and comprehensive communication with the clinical team (Table 2). When specifically asked what more help they might require from their clinical team (OEQ 2), 13 participants (12%) expressed a desire for additional support with treatment-related, psychological, practical, and diagnostic concerns (Supplementary file 4). Finally, when asked to spontaneously identify their top three concerns (OEQ 3), responses from 40 participants (38%) revealed concerns around future response to treatment, running out of treatment options, cancer relapse, declining health, dying, and impact on family (Fig. 3).

**Table 2:** Additional concerns spontaneously reported in response to the open-ended question: *Are there any other issues that we have not mentioned but are important to you?* (*N* = 18)

Category	Quote
Treatment-related issues	*Staying on immunotherapy past 2 years. [SURV06]*
	*I am on Pazopanib, I would like to know how long it works for, the statistics. [SURV105]*
	*Taking treatment every single day causes depression. Better treatments are needed. I often wonder if manufacturers would use the treatments they make? [SURV78]*
	*I find it depressing that current treatments don’t work for very long (disease becomes resistant). Lots of side effects but very little effective time. We need better treatments and I feel kidney cancer isn’t as popular as other cancers, therefore the research is slow. [SURV94]*
	*I am concerned that drugs may be difficult to source if the shortages of everything crisis keeps getting worse. [SURV106]*
	*Sleep disturbed as I have to frequently get up during the night. [SURV05]*
	*My first cancer treatment was immunotherapy. This did me some considerable damage. [SURV07]*
Health system access	*I worry a lot about access to the healthcare system due to system overload, long wait lists. [SURV77]*
	*Added complication to avoid COVID. [SURV04]*
Practical issues	*Lost my driving licence due to brain tumour which I have had treated. Hope to get it back but is a problem in the meantime. [SURV03]*
	*Travel to Oncology Centre. [SURV88]*
Support/advice	*Contact/discussions about treatment with other patients is often helpful and gives support. [SURV87]*
	*How to prevent future illness. [SURV98]*
	*When treatment is stopped because of not working. [SURV101]*
	*Access to advice and emotional well-being. [SURV103]*
Communication with the clinical team	*I would like to discuss my situation with professionals, what can I do in next step? I want to get well. [SURV88]*
	*When first diagnosed, I was totally unprepared and wish I knew what I know now about RCC. I would rather have been overwhelmed with correct valid information about RCC rather than given a generic Macmillan pack and left to search Dr Google for information (which may not be accurate). [SURV86]*
	*I worry that my Oncology Unit don’t really understand my treatment & the side effects. Sometimes I seem to know more than they do. [SURV95]*

### Predictors of wellbeing

Neither gender nor time since diagnosis was linked to any of the wellbeing domains (all *p* > 0.05) (Table 3). Each one SD increase in age was linked to a 0.46 SD increase in physical wellbeing (95% CI 0.26, 0.67), a 0.54 SD increase in emotional wellbeing (95% CI 0.35, 0.74), a 0.37 SD increase in FACT-BRM TOI score (95% CI 0.15, 0.60), a 0.51 SD increase in FACT-G score (95% CI 0.31, 0.72), and a 0.50 SD increase in FACT-BRM scores (95% CI 0.29, 0.71).

**Table 3:** Multivariate linear regression models of FACT subscale and total scores

Covariate	Physical wellbeing							Emotional wellbeing					
	*b*	SE	β		*t*	95% CI^		*b*		SE	β	*t*	95% CI^
Constant	10.76	2.56			4.20**			4.86		1.87		2.59*	
Gender (ref: male)	− 0.83	1.09	− 0.70		− 0.76	− 0.25, 0.11		− 0.27		0.80	− 0.03	− 0.33	− 0.21, 0.15
Age (years)	0.20	0.04	0.46		4.49**	0.26, 0.67		0.17		0.03	0.54	5.45**	0.35, 0.74
Time since diagnosis (months)	− 0.01	0.01	− 0.11		− 0.99	− 0.34, 0.11		0.01		0.01	0.08	0.71	− 0.14, 0.30
TT (ref: CT)	− 3.83	1.55	− 0.30		− 2.47*	− 0.54, − 0.06		− 0.40		1.13	− 0.04	− 0.35	− 0.28, 0.19
IOT (ref: CT)	− 1.93	1.49	− 0.16		− 1.30	− 0.40, 0.08		0.08		1.09	0.01	0.07	− 0.23, 0.24
HT (ref: CT)	− 2.46	2.21	− 0.11		− 1.11	− 0.31, 0.09		0.94		1.62	0.06	0.58	− 0.14, 0.25
RT (ref: CT)	− 10.58	2.83	− 0.45		− 3.74**	− 0.68, − 0.21		− 6.75		2.07	− 0.38	− 3.26*	− 0.61, − 0.15
Model statistics	Overall *R*^2^ = 0.34; adjusted *R*^2^ = 0.29; *F* (7,85) = 6.27**							Overall *R*^2^ = 0.38; adjusted *R*^2^ = 0.33; *F* (7,85) = 7.34**					
Covariate	Social/family wellbeing							Functional wellbeing					
	*b*	SE	β		*t*	95% CI^		*b*		SE	β	*t*	95% CI^
Constant	9.70	2.57			3.77**			13.88		2.35		5.91**	
Gender (ref: male)	0.49	1.10	0.04		0.44	− 0.15, 0.24		− 0.72		1.01	− 0.07	− 0.71	− 0.27, 0.13
Age (years)	0.06	0.04	0.15		1.38	− 0.07, 0.38		− 0.04		0.04	− 0.11	− 0.96	− 0.33, 0.11
Time since diagnosis (months)	0.00	0.01	0.01		0.06	− 0.24, 0.26		0.01		0.01	0.13	1.06	− 0.12, 0.38
TT (ref: CT)	5.10	1.56	0.43		3.28*	0.17, 0.70		3.20		1.42	0.30	2.26*	0.04, 0.56
IOT (ref: CT)	3.40	1.50	0.30		2.27*	0.04, 0.56		4.08		1.37	0.39	2.98*	0.13, 0.65
HT (ref: CT)	2.66	2.22	0.13		1.20	− 0.09, 0.35		4.02		2.02	0.22	1.99	0.00, 0.43
RT (ref: CT)	7.05	2.85	0.32		2.48*	0.06, 0.58		8.86		2.60	0.44	3.41**	0.18, 0.70
Model statistics	Overall *R*^2^ = 0.22; adjusted *R*^2^ = 0.16; *F* (7,85) = 3.41*							Overall *R*^2^ = 0.23; adjusted *R*^2^ = 0.17; *F* (7,85) = 3.61*					
Covariate	FACT-BRM TOI							FACT-G					
	*b*	SE	β		*t*	95% CI^		*b*		SE	β	*t*	95% CI^
Constant	49.60	6.43			7.72**			40.15		4.81		8.35**	
Gender (ref: male)	− 4.28	2.76	− 0.16		− 1.55	− 0.35, 0.04		− 2.00		2.07	− 0.09	− 0.97	− 0.27, 0.09
Age (years)	0.36	0.11	0.37		3.30*	0.15, 0.60		0.41		0.08	0.51	4.96**	0.31, 0.72
Time since diagnosis (months)	− 0.00	0.04	− 0.01		− 0.07	− 0.26, 0.24		− 0.00		0.03	− 0.01	− 0.06	− 0.24, 0.22
TT (ref: CT)	− 3.85	3.88	− 0.13		− 0.99	− 0.40, 0.13		3.85		2.90	0.16	1.33	− 0.08, 0.40
IOT (ref: CT)	− 1.80	3.75	− 0.06		− 0.48	− 0.33, 0.20		4.91		2.81	0.21	1.75	− 0.03, 0.45
HT (ref: CT)	− 2.01	5.53	− 0.04		− 0.36	− 0.26, 0.18		4.96		4.14	0.12	1.20	− 0.08, 0.32
RT (ref: CT)	− 15.59	7.12	− 0.29		− 2.19*	− 0.55, − 0.03		− 0.24		5.33	− 0.01	− 0.04	− 0.24, 0.23
Model statistics	Overall *R*^2^ = 0.22; adjusted *R*^2^ = 0.15; *F* (7,85) = 3.30*							Overall *R*^2^ = 0.35; adjusted *R*^2^ = 0.29; *F* (7,85) = 6.39**					
Covariate	FACT-BRM												
	*b*			SE			β		*t*			95% CI^	
Constant	64.55			7.48					8.63**				
Gender (ref: male)	− 4.33			3.21			− 0.13		− 1.35			− 0.31, 0.06	
Age (years)	0.60			0.13			0.50		4.72**			0.29, 0.71	
Time since diagnosis (months)	0.00			0.04			0.01		0.04			− 0.23, 0.24	
TT (ref: CT)	0.76			4.51			0.02		0.17			− 0.23, 0.27	
IOT (ref: CT)	1.39			4.37			0.04		0.32			− 0.21, 0.29	
HT (ref: CT)	1.51			6.44			0.02		0.23			− 0.18, 0.23	
RT (ref: CT)	− 14.80			8.28			− 0.22		− 1.79			− 0.46, 0.02	
Model statistics	Overall *R*^2^ = 0.32; adjusted *R*^2^ = 0.26; *F* (7,85) = 5.61**												

*FACT-BRM* Functional Assessment of Cancer Therapy-Biologic Response Modifier, *TOI* Trial Outcome Index, *FACT* Functional Assessment of Cancer Therapy, *FACT-G* Functional Assessment of Cancer Therapy-General, *SE* standard error, *CI* confidence interval, *CT* chemotherapy, *IOT* immunotherapy, *TT* targeted therapy, *RT* radiotherapy, *HT* hormone therapy

*p < 0.05; **p < 0.001

^95% CI of β

Compared to chemotherapy, being on radiotherapy was associated with a 0.45 SD decrease in physical wellbeing (95% CI − 0.68, − 0.21), a 0.38 SD decrease in emotional wellbeing (95% CI − 0.61, − 0.15), and a 0.29 SD decrease in FACT-BRM TOI scores (95% CI − 0.55, − 0.03). However, compared to chemotherapy, being on radiotherapy was linked to a 0.32 SD increase in social/family wellbeing (95% CI 0.06, 0.58) and a 0.44 SD increase in functional wellbeing (95% CI 0.18, 0.70). Compared to chemotherapy, being on targeted therapy was linked to a 0.30 SD decrease in physical wellbeing (95% CI − 0.54, − 0.06) (Table 3).

## Discussion

In this survey, a sizeable proportion of participants were burdened with limitations to their ability to work, possibly linked to problems with fatigue, weakness, and treatment side effects. Feeling restricted workwise is a particularly notable finding given that over 80% of our sample were people of working age and therefore still urged to provide for their families and contribute to society. Irrespective of the age factor, a desire to be active and feel useful can be tied to keeping up with normality [23], which can be hampered by issues like functional weakness, reduced cognitive performance, and issues with mobility that our sample reported. These issues can be considered together with the resultant financial impact, which can further complicate life particularly for those already in a financially challenged situation and bring about social isolation that might lead to withdrawal and depressed mood. With rich data across all domains of wellbeing, it would be intriguing to investigate ‘problem clusters’ akin to the well-known concept of ‘symptom clusters’ [24]. Problems seldom come up in isolation, and problems of diverse nature usually co-occur for most people [25–27], including patients with advanced RCC. Problem clusters can become particularly pertinent as new combination therapies are approved for advanced RCC to inform value-based health through patient-perceived benefit.

Using a PROM in practice can quantify the burden of cancer-related issues [8]. It will no doubt help identify patients who need urgent support and those who are keeping well. At the same time, it can identify a likely sizeable proportion of patients who experience issues of moderate intensity—as shown in the present study. While some patients in this ‘moderate intensity’ subgroup will succeed in self-managing or seeking help early, others will transition to higher levels of concern or burden, particularly where patient activation levels are insufficient to address the underlying problem [28]. In our survey, many patients reported ‘moderate intensity’ problems within several areas of functioning and wellbeing. Such issues may be concurrent and burdensome simply because they are persistent without necessarily changing in intensity, which puts treatment outcomes at risk due to patient non-adherence or early discontinuation [29–31]. One example is cancer-related fatigue, which can be present at low-to-moderate levels but still troubling because of being persistent over long periods [32] and as such adversely impacting independence and activities of daily living [33]. Such ‘moderate intensity’ issues require active screening, ongoing monitoring, and triage for intervention, which electronic PROM systems can nowadays offer, allowing clinical teams to review automatically generated summaries of PRO data for enhanced decision-making [31, 34, 35].

Our survey sample expressed issues with troublesome side effects, accessibility to novel treatments, and availability of treatments that work for them, as has been identified in previous research [7]. Despite persistent and at least moderately burdensome side effects (e.g. fatigue, mouth sores, diarrhoea, chills, pain in joints), patients will often dwell on the inner dilemma of quality of life over life extension [36]. Some might still ask for a new treatment option when all else fails, while others will question the point of continuing and explore end-of-life care more consciously [36, 37]. Three quarters of our sample worried about a worsening illness, for which effective treatment is no longer available. Giles et al. (2022) reported similar levels of anxiety and fear of recurrence or dying among 1983 survey participants with RCC [38]. Consciously letting go of treatment while managing to cope is an extremely hard mental process [39], which not every patient can initiate or undergo. Preparing for palliative and end-of-life care was voiced as a concern for several of our sample, which might reflect the struggle to decide what to do for the best. This decision may be tied to the anticipated impact of an aggravating illness on family members and the patients’ desire to find ways to combine good quality care while they relieve the pressure on the family [36]. Drivers behind such decisions are highly personalised [37]. Younger patients and younger families might struggle more with this kind of decision-making [36, 37] (possibly explaining poorer wellbeing among younger participants in our analyses), while personality traits, educational attainment, and life goals play an additional key role [36, 37, 40]. However hard a decision to transition to palliative and end-of-life care is, the thought around the future of a patient’s loved ones will persist [37], and our sample clearly expressed this as a priority concern, which requires open discussion and support.

Emerging evidence indicates that the involvement of the entire multidisciplinary team (MDT) in discussions about an individual’s health status is linked to improved survival through enhanced decision-making [41]. Part of this relies on responding to PROs flagged up as deteriorating or of concern [42] via multidisciplinary action [43]. PRO-driven models of care can be further enhanced where artificial intelligence brings together PRO, behavioural, and clinical data (see for instance [44]) to provide MDTs with a fuller picture of a patient’s health status. Crucially, encouraging prioritisation of PROs intensifies a person-centred and ‘What matters to you?’ approach. In our study, we gave participants an opportunity to freely express what came to mind first as a priority concern. While problems can certainly be identified as severe, burdensome, or even debilitating, patients should be able to prioritise them to access maximum benefit from available supportive care interventions [45–48]. Such an approach can help increase the sense of personhood in the care service, where people are treated with a view to preserve their dignity [49].

Person-centred care is the first among seven standards set by MASCC and ASCO in 2024 to optimise survivorship care and health outcomes for people affected by advanced or metastatic cancer [50]. Importantly, person-centred care should be coordinated/integrated (standard 2) and accessible/equitable (standard 5). Integration of electronic systems to allow standardised PROM-based patient assessments, a conscious ‘What matters to you?’ approach by the MDT, and an established network of referral pathways can (among other mechanisms [51]) facilitate coordination and management of high intensity patient-reported issues and concerns within and beyond the healthcare service. In the community, support groups and peer support groups via patient organisations (e.g. International Kidney Cancer Coalition, https://ikcc.org/) can provide a means to share thoughts and perhaps find therapeutic benefit in an environment where people share similar experiences [52], particularly for ‘moderate intensity’, yet persistent and troubling, psychosocial issues. Optimal care is unachievable without proper attention to patient accessibility and equity. Person-centred care means tackling the individual barriers that prevent a patient from accessing and benefiting from available support mechanisms [53, 54]. Men, patients with less common cancers, patients who are less confident with technology, minority groups, patients in socioeconomically deprived areas, patients from geographically remote areas, and those with a disability are less likely to access and/or engage with cancer support services [55–58], and therefore, a focussed effort is critical to offer diverse, needs-specific and ability-adjusted options for unhindered and value-based engagement and participation in one’s own care [53, 54].

### Limitations

Although we relied on engagement of a worldwide network of patient support organisations to disseminate information about the survey and invite patients to take part, our patient sample comprised primarily people from English-speaking countries as the survey was only available in English, so our sample is not representative of an international sample. It is possible that the results and opinions expressed by our sample do not reflect those of members of the public in non-English-speaking countries. Our findings will need to be validated in other contexts to ensure these results are generalisable. Reports on financial distress might have been overemphasized in this sample because of the inclusion of a sizeable proportion of participants from the USA. Given the nature of the healthcare system in the USA, these patients might have experienced greater financial toxicity [59] compared to other countries. The survey was cross-sectional so gave only one snapshot of people’s experiences. We designed an online survey, which may limit the views of patients who may not access or feel comfortable with technology. Equally, an online survey and recruitment via social media might explain why our sample was, on average, younger (mean 43.9 years) compared to RCC, most frequently being diagnosed between the ages of 65 and 74. Response rates to OEQ only ranged from 12 to 38% of the total sample, which could be pointing to two possible issues, i.e. most participants were affected by survey fatigue and/or most participants had no specific issues to raise. Although we cannot affirm the exact reasons, caution should be exercised when interpreting these results. We relied on patients to self-identify as diagnosed with advanced RCC, which cannot rule out the possibility of including participants with non-advanced RCC. This might explain why 21% of participants reported being on chemotherapy, although this is not standard for advanced RCC. Finally, we acknowledge that, in regression analyses, the effective sample size for estimating the effect of radiotherapy is quite small. This may lead to unstable and imprecise coefficient estimates for that variable, which can impact the validity of the model. As such, the reader should exercise caution when considering the possible effects of radiotherapy on wellbeing from our analysis.

## Conclusions

Relying on PROs, we were able to reveal the impact on several interrelated areas in the context of advanced RCC. Concern around the ability to work, worries about a worsening health status, impact on family, and urinary frequency emerged as important issues in this sample. Further concerns about future response to treatment, running out of treatment options, cancer relapse, and dying were also expressed. Greater focus should be placed on developing further PROs in advanced RCC, particularly aiming to understand how patients and families experience the dynamic nature of PROs, and what internal and external factors impact the decisions they make to seek help when new problems appear or when previous problems become more or less intense. It is important to build on studies such as this to consider the experience of advanced RCC on people’s everyday lives and ensure that services are responding adequately to changing needs.

## Supplementary Information

Below is the link to the electronic supplementary material.

Supplementary file1 (DOCX 32 KB)

## Data Availability

The data that support the findings of this study are not openly available due to reasons of sensitivity and are available from the corresponding author upon reasonable request. Data are located in controlled access data storage at the University of Glasgow.
