# Supplementary material for: Wellbeing status and priority concerns of patients with advanced renal cell carcinoma: results of the EONS PROMs project international online survey
Source: Support Care Cancer. 2025 Jun 3;33(6):531. doi: 10.1007/s00520-025-09585-5 (PMC12130110; doi:10.1007/s00520-025-09585-5)
Supplement: Supplementary file 1 — Supplementary file1 (DOCX 32 KB) [file 520_2025_9585_MOESM1_ESM.docx]

**SUPPLEMENTARY FILES**

**Supplementary file 1.** The 30 survey items additional to the FACT-BRM.

1. I feel fatigued
2. I have been short of breath
3. I have vomited
4. I am losing weight
5. I have diarrhea (diarrhoea)
6. I have mouth sores
7. I can swallow naturally and easily
8. I have been coughing
9. I have changes in the skin in my hands and/or feet
10. My heart is racing (I have palpitations)
11. I have had blood in my urine
12. I have trouble controlling my urine
13. I urinate more frequently than usual
14. I have trouble bringing to mind words that I want to use while talking to someone
15. I am stressed
16. I feel anxious
17. I feel like I am losing my dignity
18. I feel frightened
19. I feel lonely
20. I feel helpless
21. I have trouble doing strenuous activities (like carrying a heavy shopping bag or a suitcase)
22. I have trouble walking
23. I need help with basic self-care activities (e.g. eating, dressing, washing myself or using the toilet)
24. I am concerned about end-of-life care
25. I feel weak to function.
26. I worry about the practical support my partner or family gets
27. I worry about the psychological support my partner or family gets
28. My treatment meets my expectations
29. I worry about getting access to cancer screenings
30. I am able to attend or travel to appointments

**Supplementary file 2.** Descriptive statistics of FACT-BRM subscales and total scores, including Cronbach’s alpha as a measure of internal consistency within subscale items of the FACT-BRM.

| N=105 | **N ítems** | **Mean** | **Median** | **SD** | **Min** | **Max** | **Alpha**** |
| --- | --- | --- | --- | --- | --- | --- | --- |
| Physical wellbeing (PWB) | 7 | 14.9 | 16.0 | 6.0 | 3.0 | 28.0 | 0.88 |
| Social-family wellbeing (SWB) | 7 | 16.2 | 15.0 | 5.6 | 0.0 | 28.0 | 0.84 |
| Emotional wellbeing (EWB) | 6 | 12.3 | 12.0 | 4.4 | 0.0 | 21.0 | 0.76 |
| Functional wellbeing* (FWB) | 7 | 15.2 | 14.0 | 5.1 | 0.0 | 26.0 | 0.82 |
| Additional concerns – BRM physical* | 7 | 14.6 | 14.0 | 5.3 | 0.0 | 27.0 | 0.81 |
| Additional concerns – BRM mental* | 6 | 12.3 | 12.0 | 4.1 | 1.0 | 21.0 | 0.79 |
| FACT-BRM TOI* | n/a | 56.9 | 55.8 | 13.2 | 19.0 | 97.0 | n/a |
| FACT-G Total* | n/a | 58.5 | 56.0 | 10.9 | 37.0 | 89.0 | n/a |
| FACT-BRM Total* | n/a | 85.3 | 81.5 | 16.4 | 49.0 | 134.0 | n/a |
| **N=104; **Cronbach’s Alpha; n/a – Not applicable; SD – Standard deviation* | | | | | | | |

**Supplementary file 3.** Prevalence of problems across all domains in descending order.

|  | **Total N for item** | **A major problem** | **Somewhat of a problem** | **Not a problem** |
| --- | --- | --- | --- | --- |
| Being able to work (FWB) | 104 | 46% | 27% | 27% |
| Worrying condition will get worse (EWB) | 105 | 45% | 32% | 23% |
| Worrying about psychological support for family* | 104 | 44% | 33% | 23% |
| Urinating more frequently than usual* | 104 | 43% | 35% | 22% |
| Feeling weak to function* | 104 | 39% | 36% | 25% |
| Feeling helpless* | 103 | 39% | 29% | 32% |
| Getting tired easily (BRM-Physical) | 104 | 39% | 28% | 33% |
| Mouth sores* | 104 | 38% | 35% | 28% |
| Bothered by side effects of treatment (PWB) | 105 | 38% | 34% | 28% |
| Diarrhea* | 104 | 38% | 32% | 31% |
| Trouble doing strenuous activities* | 104 | 38% | 22% | 40% |
| Getting annoyed easily (BRM-Mental) | 104 | 37% | 36% | 28% |
| Pain in joints (BRM-Physical) | 104 | 37% | 33% | 31% |
| Feeling lonely* | 104 | 37% | 32% | 32% |
| Bothered by sweating (BRM-Physical) | 104 | 37% | 28% | 36% |
| Concerned about end-of-life care* | 104 | 37% | 27% | 37% |
| Emotional ups and downs (BRM-Mental) | 104 | 36% | 40% | 24% |
| Trouble bringing to mind words* | 104 | 36% | 35% | 30% |
| Trouble remembering things (BRM-Mental) | 104 | 36% | 29% | 36% |
| Feeling fatigued* | 104 | 36% | 26% | 38% |
| Feeling nervous (EWB) | 105 | 35% | 35% | 30% |
| Trouble walking* | 104 | 35% | 35% | 31% |
| Trouble concentrating (BRM-Mental) | 104 | 35% | 24% | 41% |
| Bothered by the chills (BRM-Physical) | 104 | 34% | 32% | 35% |
| Feeling weak all over (BRM-Physical) | 104 | 34% | 31% | 36% |
| Satisfied with sex life (SWB) | 100 | 34% | 28% | 38% |
| Work is fulfilling (FWB) | 103 | 33% | 36% | 31% |
| Forced to spend time in bed (PWB) | 105 | 33% | 31% | 35% |
| Feeling ill (PWB) | 104 | 33% | 31% | 37% |
| Having trouble controlling urine* | 104 | 33% | 29% | 38% |
| Changes in the skin of hands / feet* | 104 | 32% | 33% | 36% |
| Trouble meeting the needs of family (PWB) | 105 | 32% | 31% | 36% |
| Feeling frightened* | 104 | 32% | 26% | 42% |
| Worrying about dying (EWB) | 105 | 31% | 39% | 30% |
| Lack of energy (PWB) | 105 | 31% | 34% | 34% |
| Worrying about getting access to cancer screening* | 104 | 31% | 33% | 37% |
| Palpitations* | 104 | 31% | 31% | 38% |
| Swallowing naturally and easily* | 104 | 31% | 21% | 48% |
| Pain (PWB) | 105 | 30% | 30% | 39% |
| Feeling close to friends (SWB) | 105 | 30% | 30% | 40% |
| Losing hope against cancer (EWB) | 105 | 30% | 30% | 39% |
| Losing weight* | 104 | 30% | 29% | 41% |
| Blood in urine* | 104 | 30% | 28% | 42% |
| Shortness of breath* | 104 | 30% | 28% | 42% |
| Worrying about practical support for family* | 104 | 30% | 27% | 43% |
| Feeling anxious* | 104 | 29% | 35% | 37% |
| Bothered by fevers (BRM-Physical) | 104 | 29% | 31% | 40% |
| Getting depressed easily (BRM-Mental) | 104 | 29% | 30% | 41% |
| Getting support from friends (SWB) | 105 | 29% | 30% | 42% |
| Vomiting* | 104 | 29% | 24% | 47% |
| Appetite (BRM-Physical) | 104 | 28% | 36% | 37% |
| Losing dignity* | 104 | 28% | 34% | 38% |
| Satisfied with coping with illness (EWB) | 104 | 28% | 32% | 40% |
| Getting emotional support from family (SWB) | 105 | 28% | 28% | 45% |
| Treatment meets expectations* | 104 | 27% | 30% | 43% |
| Needing help with basic self-care activities* | 104 | 27% | 29% | 44% |
| Enjoying things usually doing for fun (FWB) | 104 | 26% | 32% | 42% |
| Able to enjoy life (FWB) | 104 | 25% | 30% | 45% |
| Feeling stressed* | 104 | 25% | 24% | 51% |
| Feeling sad (EWB) | 104 | 24% | 26% | 50% |
| Sleeping well (FWB) | 104 | 23% | 36% | 41% |
| Content with quality of life right now (FWB) | 104 | 23% | 36% | 41% |
| Coughing* | 104 | 22% | 34% | 44% |
| Nausea (PWB) | 104 | 19% | 25% | 56% |
| Feeling motivated to do things (BRM-Mental) | 104 | 16% | 41% | 42% |
| Satisfied with family communication about the illness (SWB) | 105 | 16% | 32% | 51% |
| Feeling close to partner (SWB) | 104 | 14% | 33% | 53% |
| Family has accepted the illness (SWB) | 105 | 11% | 32% | 56% |
| Able to attend or travel to appointments* | 104 | 10% | 35% | 56% |
| Accepting own illness (FWB) | 104 | 8% | 42% | 50% |
| **Item part of the 30 survey items additional to the FACT-BRM.*  *PWB – Physical wellbeing; SWB – Social-family wellbeing; EWB – Emotional wellbeing; FWB – Functional wellbeing; BRM-Physical – FACT-BRM Additional concerns: Physical; BRM mental – FACT-BRM Additional concerns: Mental.* | | | | |

**Supplementary file 4.** Need for help spontaneously expressed in response to the open-ended question: *Is there any help from your clinical team that you need but have not received yet?* (N=13)

| **Category** | **Quote** |
| --- | --- |
| Treatment-related | *Don’t have access to immune and targeted therapy. [SURV88]* |
|  | *I would like them to be more aware of my treatment & side effects. [SURV95]* |
|  | *What can they do? We need a cure for kidney cancer and better treatments. It isn't something they can help with. [SURV78]* |
|  | *An overview of results of trials and scientific progress would give hope for the future. [SURV87]* |
| Psychological support | *Regular check-ins regarding psychological support, coping. [SURV77]* |
|  | *Mental health support for my young children. [SURV99]* |
| Practical support | *Nursing care at home. [SURV90, SURV05]* |
|  | *Dietary advice. [SURV04]* |
| Diagnostic imaging results | *CT scans are very slow to come through and are never on time. [SURV07]* |
|  | *Scans take a long time to come through. [SURV83]* |
|  | *Bone and brain scans are long overdue. CT scans are always months late. [SURV103]* |
|  | *I never see the scan images which I always saw before I moved. [SURV104]* |
